# Translating Clinical Findings into Knowledge in Drug Safety Evaluation - Drug Induced Liver Injury Prediction System (DILIps)

**DOI:** 10.1371/journal.pcbi.1002310

**Published:** 2011-12-15

**Authors:** Zhichao Liu, Qiang Shi, Don Ding, Reagan Kelly, Hong Fang, Weida Tong

**Affiliations:** 1 Division of Systems Biology, National Center for Toxicological Research, United States Food and Drug Administration, Jefferson, Arizona, United States of America; 2 ICF International Company at FDA's National Center for Toxicological Research, Jefferson, Arizona, United States of America; National University of Singapore, Singapore

## Abstract

Drug-induced liver injury (DILI) is a significant concern in drug development due to the poor concordance between preclinical and clinical findings of liver toxicity. We hypothesized that the DILI types (hepatotoxic side effects) seen in the clinic can be translated into the development of predictive *in silico* models for use in the drug discovery phase. We identified 13 hepatotoxic side effects with high accuracy for classifying marketed drugs for their DILI potential. We then developed *in silico* predictive models for each of these 13 side effects, which were further combined to construct a DILI prediction system (DILIps). The DILIps yielded 60–70% prediction accuracy for three independent validation sets. To enhance the confidence for identification of drugs that cause severe DILI in humans, the “Rule of Three” was developed in DILIps by using a consensus strategy based on 13 models. This gave high positive predictive value (91%) when applied to an external dataset containing 206 drugs from three independent literature datasets. Using the DILIps, we screened all the drugs in DrugBank and investigated their DILI potential in terms of protein targets and therapeutic categories through network modeling. We demonstrated that two therapeutic categories, anti-infectives for systemic use and musculoskeletal system drugs, were enriched for DILI, which is consistent with current knowledge. We also identified protein targets and pathways that are related to drugs that cause DILI by using pathway analysis and co-occurrence text mining. While marketed drugs were the focus of this study, the DILIps has a potential as an evaluation tool to screen and prioritize new drug candidates or chemicals, such as environmental chemicals, to avoid those that might cause liver toxicity. We expect that the methodology can be also applied to other drug safety endpoints, such as renal or cardiovascular toxicity.

## Author Summary

Translational research involves utilization of clinical data to address challenges in drug discovery and development. The rationale behind this study is that the side effects observed in clinical trial and post-marketing surveillance can be translated into a screening system for use in drug discovery. As a proof-of-concept study, we developed an *in silico* system based on 13 hepatotoxic side effects to predict drug-induced liver injury (DILI), which is one of the most frequent causes of drug failure in clinical trial and withdrawal from post-marketing application, and also one of the most difficult clinical endpoints to predict from preclinical studies. We first identified 13 types of liver injury which yielded high prediction accuracy to distinguish drugs known to cause DILI from these don't. To effectively apply these 13 hepatotoxic side effects to the drug discovery process for DILI, we developed *in silico* models for each of these side effects solely based on chemical structure data. Finally, we constructed a DILI prediction system (DILIps) by combining these 13 *in silico* models in a consensus fashion, which yielded >91% positive predictive value for DILI in humans. The DILIps methodology can be extended in applications for addressing other drug safety issues, such as renal and cardiovascular toxicity.

## Introduction

Drug-induced liver injury (DILI) poses a significant challenge to medical and pharmaceutical professionals as well as regulatory agencies. It is the leading cause of acute liver failure, which has a high mortality rate (30%) as treatment is limited due to the availability of livers for transplantation [1]. Although many dangerous drugs are identified during animal testing thus protecting humans from this damage, a consortium determined that about half of the drugs that cause human hepatotoxicity were not identified as having this potential in nonclinical animal testing [2]. Many drugs have been withdrawn from the market or have received restrictions and warnings due to DILI [3]. DILI information and guidance for pharmaceutical industries has been released by regulatory agencies such as the U.S. Food and Drug Administration (FDA) (http://www.fda.gov/downloads/Drugs/GuidanceComplianceRegulatoryInformation/Guidances/UCM174090.pdf), European Medicines Agency (EMA) (www.ema.europa.eu/pdfs/human/swp/15011506en.pdf) and Health Canada (http://www.hc-sc.gc.ca/dhp-mps/alt_formats/pdf/consultation/drug-medic/draft_ebauche_hepatotox_guide_ld-eng.pdf), highlighting both the significance and difficulties in DILI research. In the FDA, the Critical Path Initiative identified DILI as a key area of focus in a concerted effort to broaden the agency's knowledge for better evaluation tools and safety biomarkers (http://www.fda.gov/ScienceResearch/SpecialTopics/RegulatoryScience/ucm228131.htm).

Determining the potential for a drug candidate to cause DILI in humans is a challenge. First, the standard pre-clinical animal studies do not effectively predict DILI events in humans. In one notorious example, five subjects in a phase 2 clinical trial experienced fatal hepatotoxicity induced by fialuridine, an investigational nucleoside analogue that showed no liver damage in animal studies [4]. Out of 221 pharmaceuticals, the overall concordance of liver toxicity in humans and experimental animals is as low as 55%, which is in sharp contrast with the concordance of other target organs such as the hematological (91%), gastrointestinal (85%), and cardiovascular (80%) systems [2]. Secondly, even well-controlled clinical trials fail to accurately predict post-marketing DILI events. The main reason for this is the statistical power of the trials – the risk of severe DILI of an idiosyncratic nature is very low per exposed subject, while clinical trials are usually carried out with only several thousand patients [5], rendering them significantly underpowered to predict rare DILI events.

To enhance the predictability of DILI, novel approaches have been explored by many researchers. Notable examples include (a) development of new DILI biomarkers [6], (b) introduction of high-content screening [7], (c) adoption of more sensitive animal models [8], [9], [10], and (d) utilization of toxicogenomics [11]. Most of these investigations are focused on developing biomarkers using either animal or *in vitro* models for predicting DILI in humans. This still would involve synthesis of the drug and elaborate testing. An *in silico* approach could inform chemists at the earliest point in the drug discovery pipeline and enable them to select the best chemical structures.

We hypothesized that there exists a distinct set of liver side effects that can be used to characterize the DILI risk of drugs in humans. We identified 13 types of hepatotoxicity (hepatotoxic side effects or HepSEs) from the organ levels of *hepatobiliary disorders* in the Medical Dictionary for Regulatory Activities (MedDRA) ontology (http://www.meddramsso.com/). We found that these 13 HepSEs can discriminate DILI drugs from non-DILI drugs with high accuracy (∼83%). Since the side effects are clinical observations obtained either from clinical trials or from post-marketing surveillance with limited utility in drug discovery, we developed quantitative structure-activity relationship (QSAR) models for each of the HepSEs. We then constructed a DILI prediction system (DILIps) based on the 13 HepSE models with an improved prediction strategy using a “Rule of Three” (RO3) criterion (incriminated by 3 or more HepSE models). The systems were evaluated in several external test sets with performance surpassing most *in silico* models in the field. We screened the entire drug list using the DILIps and evaluated the RO3 drugs in terms of therapeutic use and drug targets.

## Results

### Identification and assessment of hepatotoxic side effects (HepSEs)

**Figure 1:**
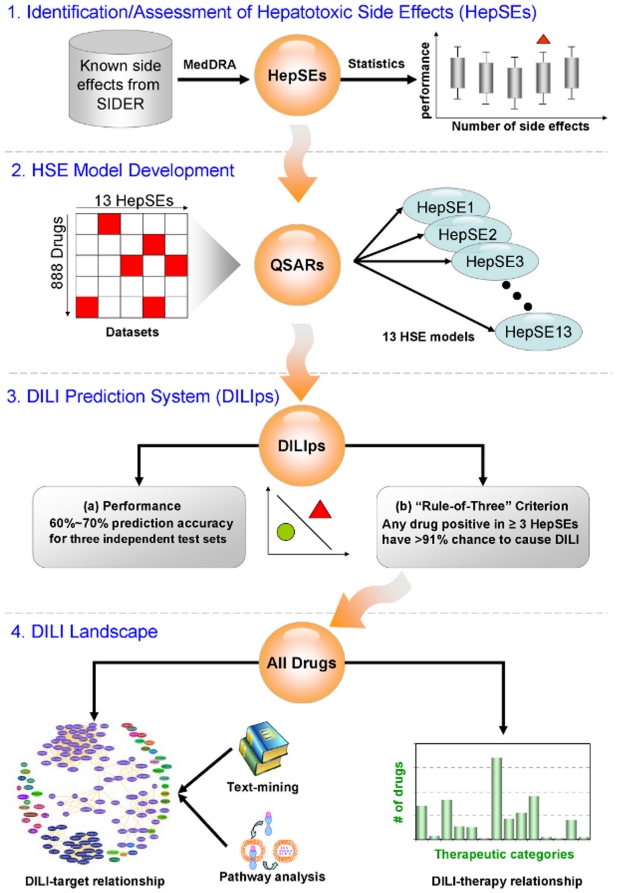
Overview of the workflow of DILIps development and its evaluation.

Figure 1 is an overview of the approach taken. First, the identification and assessment of HepSEs were performed. We used the SIDER database [12] to identify drugs and associated side effects. Out of 1450 side effects in the database, we selected only those that were caused by more than 20 drugs (an arbitrary cut-off). This yielded 473 side effects. The distribution of 888 drugs over 473 side effects and vice verse were depicted in Supplementary Figure S1, indicating that over 90% drugs were associated with at least 10 side effects. These side effects were then directly mapped onto low level terms of MedDRA. The terms were linked to the system organ classes (SOC) level according to the hierarchical structure of MedDRA (Supplementary Table S1) in order to determine the terms' attributes at the organ level. Finally, we considered side effects defined by the MedDRA ontology as related to the *hepatobilliary disorders* SOC term as HepSEs, and identified 13 HepSEs: *bilirubinemia, cholecystitis, cholelithiasis, cirrhosis, elevated liver function tests, hepatic failure, hepatic necrosis, hepatitis, hepatomegaly, jaundice, liver disease, liver fatty, and liver function tests abnormal*.

We evaluated these 13 HepSEs for their ability to differentiate drugs that do and do not cause DILI using the Liver Toxicity Knowledge Base Benchmark Dataset (LTKB-BD) [13] and PfizerData [14]. For both datasets, we used only the drugs that they had in common with SIDER. There are several differences between two datasets to call a drug as DILI or non-DILI (see *Materials and methods*), including (1) LTKB-BD is based on the FDA-approved drug labeling while PfizerData is according to the case reports; (2) two datasets apply different criteria for DILI assessment; and (3) drugs are grouped differently between two datasets. To obtain an objective evaluation for 13 HepSEs, we took the following actions to select DILI positive and negative drugs from two datasets: (a) in LTKB-BD [13], Most-DILI-Concern drugs were classified as positive while No-DILI-Concern drugs were classified as negative; and (b) in PfizerData [14], drugs with evidence in human toxicity were considered DILI positive while drugs with no evidence in any species were considered DILI negative. Defining a drug as causing DILI if it was positive in any of the 13 HepSEs, this approach yielded 91% and 74% accuracy for LTKB-BD and PfizerData, respectively.

**Figure 2:**
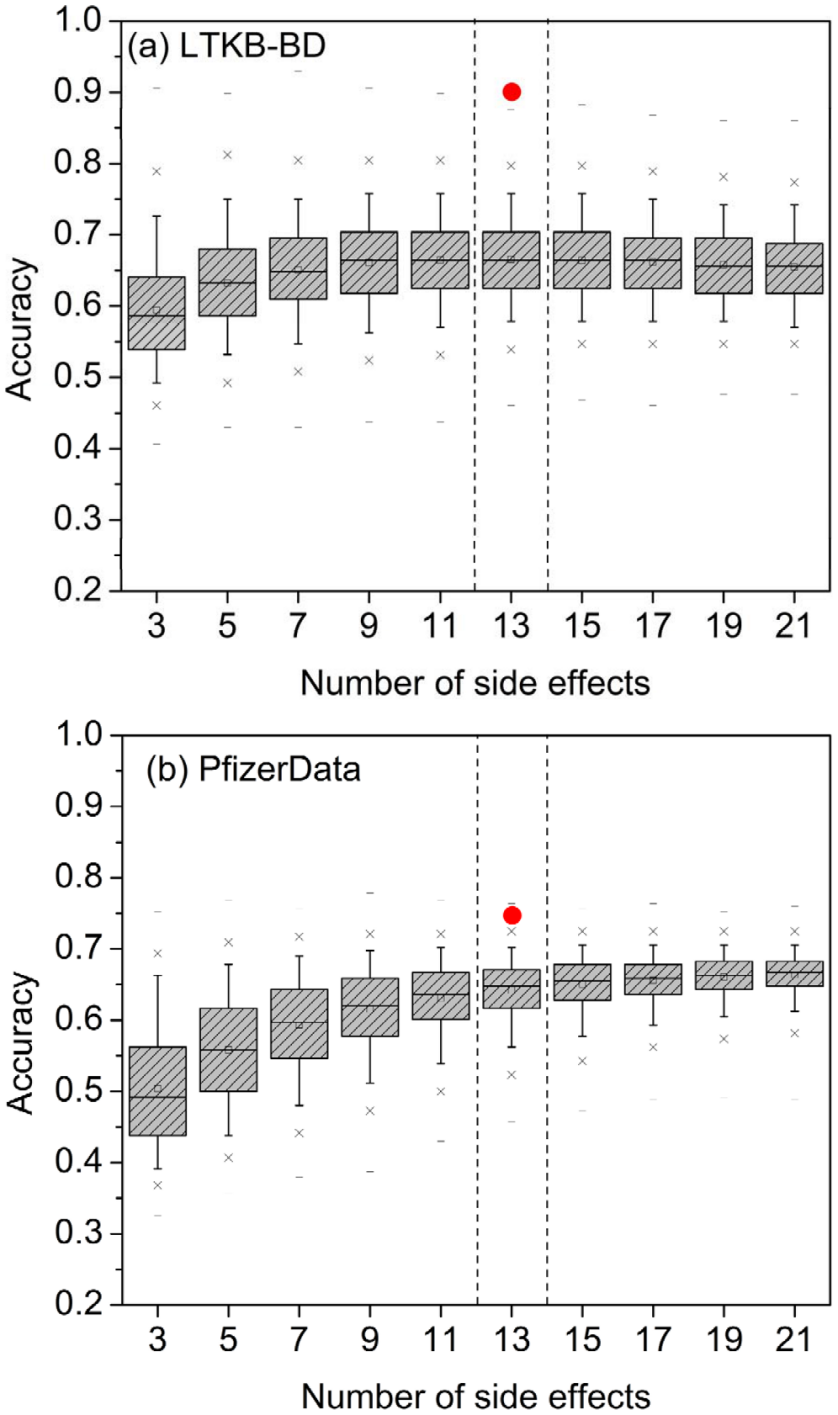
Box plot of classification accuracy with the number of selected side effects using a permutation test. (a) The test consisted of 128 drugs with a ratio of 69 DILI positives versus 59 DILI negatives in the LTKB-BD, and (b) 258 drugs with a ratio of 168 DILI positive drugs and 90 DILI negative drugs in PfizerData. Given a randomly selected number of side effects, a drug showing positive in any of the side effects was considered as a DILI positive drug. The process was repeated 20,000 times for each of the selected number of side effects. The red dot denotes the data based on the 13 HepSEs selected from the MedDRA *hepatobilliary* disorders category.

It is important to note that the 26 MedDRA SOCs are not all strictly related to human organs in a conventional sense. For example, “investigations” and “general disorders and administration site conditions” are not organs (the complete list of MedDRA SOC is available in Supplementary Table S1). Some side effects with DILI indication are resided in a SOC other than the hepatobilliary disorders. For example, the SOC of “investigations” include the “elevate liver enzyme” and “alkaline phosphatase increased”, both are conventional DILI indicators. Moreover, some side effects in the SOC of “general disorders and administration site conditions” could also be the manifestations of DILI. Thus, we conducted a permutation test with the purposes of confirming that the 13 HepSEs do in fact have significant performance over the chance to distinguish DILI drugs from non-DILI drugs. We randomly selected 3, 5,…, 21 side effects from the 473 side effect pool with each selection repeated 20,000 times. As shown in Figure 2, the classification accuracy of the 13 HepSEs, indicated by the red dot, was considerably higher than the average accuracy for each of the sets of randomly selected side effects, demonstrating that the observed classification accuracy for the 13 HepSEs was not due to chance.

### DILI prediction system (DILIps)

As illustrated in step 2 (Figure 1), QSAR models were developed for each of the 13 HepSEs to enable their use in screening new drug candidates computationally. The QSAR models developed from the drugs related to each of these 13 HepSEs had high prediction accuracy (>93%) in a leave-one-out cross-validation (LOO-CV) process (Table 1).

**Table 1:** Performance of leave-one out cross-validation for the 13 HepSE models.

HepSE models	# of drugs positive in HepSE	Accuracy	Sensitivity	Specificity
bilirubinemia	88	0.96	0.76	0.99
cholecystitis	53	0.98	0.83	0.99
cholelithiasis	60	0.98	0.75	0.99
cirrhosis	27	0.99	0.89	0.99
elevated liver function tests	29	0.99	0.76	1.00
hepatic failure	132	0.95	0.82	0.97
hepatic necrosis	56	0.97	0.91	0.97
hepatitis	254	0.93	0.91	0.94
hepatomegaly	62	0.96	0.71	0.97
jaundice	274	0.93	0.89	0.95
liver disease	42	0.98	0.74	0.99
liver fatty	22	0.99	0.82	0.99
liver function tests abnormal	111	0.95	0.83	0.97

Based on the 13 HepSE models, we further developed the DILIps (step 3 of Figure 1, left box). Using the same classification rule described above (i.e., drugs incriminated by any of the 13 HepSEs models are considered as DILI positives), we applied DILIps to three external validation sets. The validation sets of LTKB-BD and PfizerData contain drugs not used in developing the 13 HepSE models. For the O'Brien et al. dataset [15], the severe and moderate hepatotoxicity drugs were combined as DILI positive drugs while the non-toxic drugs were defined as DILI negative drugs (only the drugs not used by the 13 HepSE models were included). As summarized in Table 2, the DILIps exhibited a reasonable prediction performance for three external validation sets with the prediction accuracy between 60–70%.

**Table 2:** Performance of DILI prediction system (DILIps) on three literature datasets.

Datasets*	The number of drugs for analysis (DILI positive drugs/DILI negative drugs)	Accuracy	Sensitivity	Specificity
LTKB-BD	67/6	0.66	0.66	0.67
PfizerData	92/56	0.60	0.52	0.73
O'Brien et al.	25/15	0.70	0.56	0.93

*Only the drugs that did not overlap with the SIDER database were used.

### Development of the “Rule of Three” criterion in DILIps

Identifying the drugs of severe DILI potential with high confidence has an important application since these drugs are likely withdrawn from the market or restricted in use with black box warning (BBW) due to the serious public health concern. We assume that the number of models calling a drug causing DILI is positively correlates with the drug's severity for DILI and to the extension of the confidence to make such a call. We generated a union set based on the three validation datasets listed in Table 2. We removed three drugs having an inconsistent DILI assignment among three datasets (only three drugs were removed: moxisylyte, carbidopa and terfenadine), i.e., positive in one dataset and negative in another. This process resulted in 145 DILI positives and 63 DILI negatives (see Supplementary Table S2). We used this union set to assess how many HepSE models to be combined likely identify high risk DILI drugs (i.e., withdrawal or BBW drugs) with high positive predictive value (PPV).

**Figure 3:**
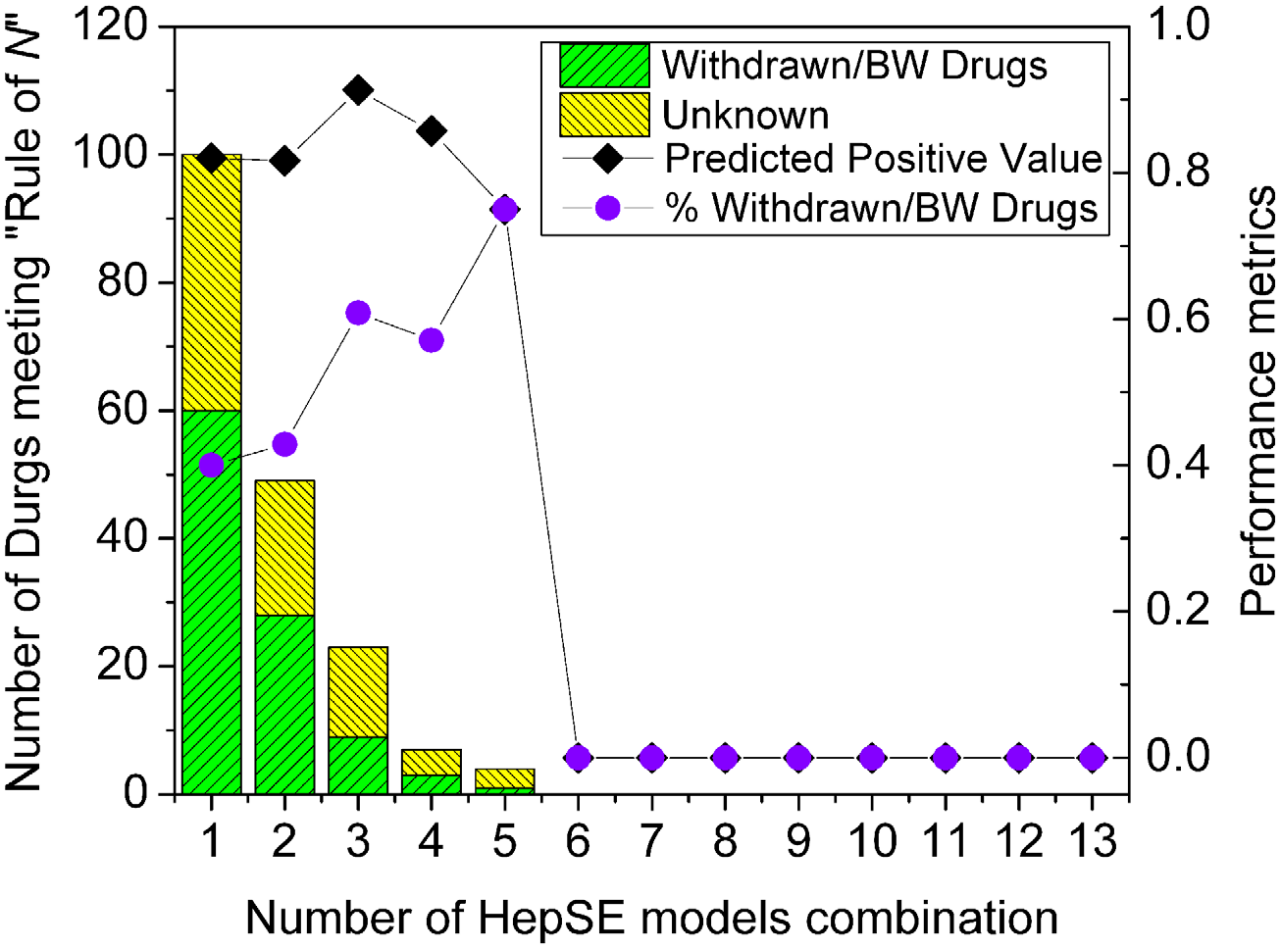
The evaluation of the “Rule of Three” (RO3). The predicted positive value, percentage of withdrawn or boxed warning (BW) drugs, and the number of drugs meeting the “Rule of *N*” for different values of *N* in the combined HepSE model.

Specifically, for each of the possible HepSE combination models requiring a drug to be incriminated by N HepSE models (“Rule of N”), we calculate PPV and the number of drugs retained by each of the HepSE combination models. As depicted in Figure 3, the PPV reaches a maximum of 91.3% when N = 3. Additionally, the percentage of high risk DILI drugs reached a local maximum at N = 3. Therefore, we established the RO3 criterion in the DILIps for identifying drugs that might cause severe DILI with high confidence (step 3 of Figure 1, right box). The number of drugs meeting the RO3 is 23, dramatically decreased from 100 (RO1) and 49 (RO2), which was expected when the optimization was tilted toward increasing PPV. In order to identify the drugs of severe DILI potential with high confidence, the trade-off was accepted in the context of an application. Therefore, the RO3 was selected to carry out further study.

### DILI potential varies for different therapeutic categories

**Figure 4:**
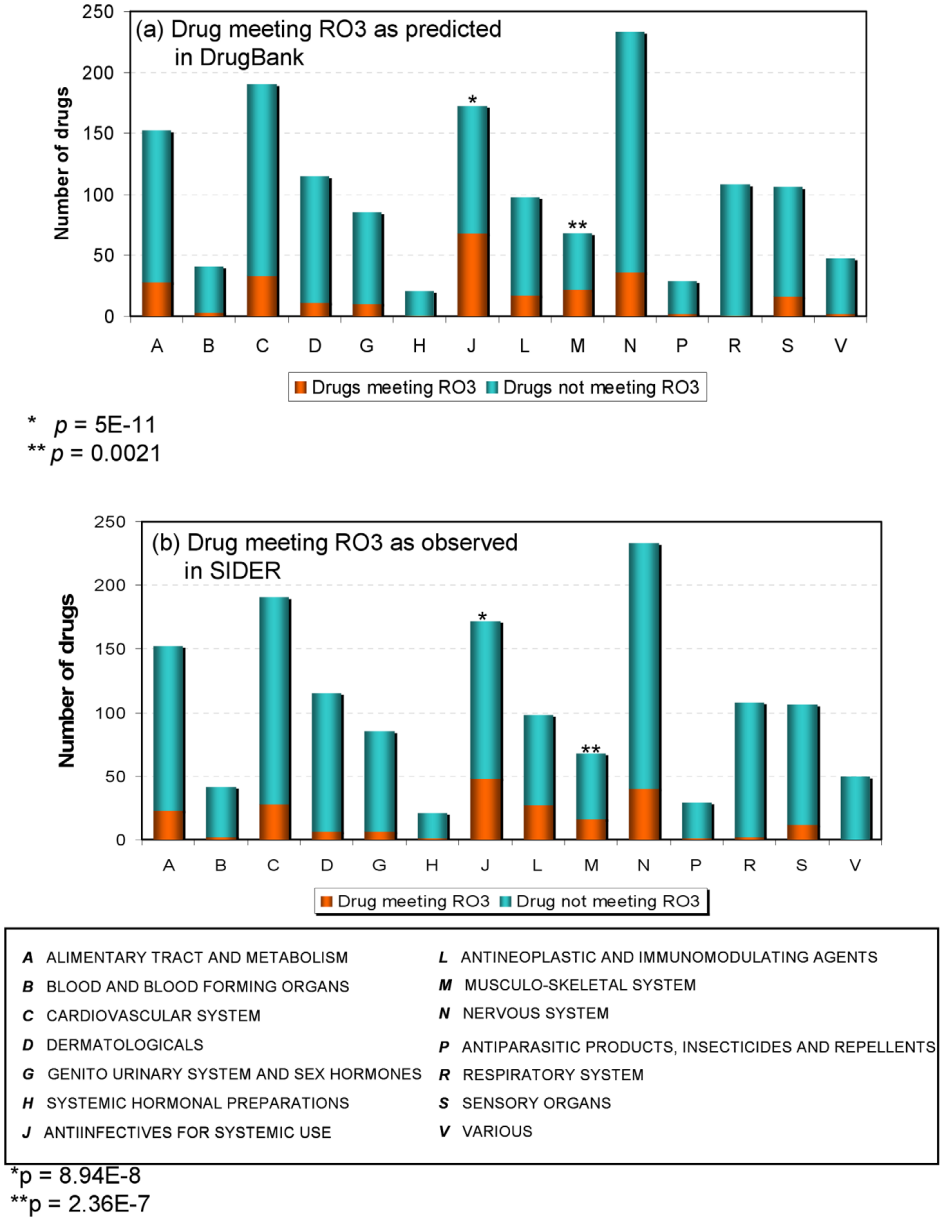
The distribution of small molecular drugs in (a) DrugBank and (b) SIDER that satisfy the “Rule of Three” (RO3) at the first level of Anatomical Therapeutic Chemical Classification System (ATC). The probability of the presence of DILI drugs is statistically significant in two therapeutic categories (J and M).

We applied the RO3 criterion to the drugs (small molecules only) in DrugBank to investigate which therapeutic categories were most likely associated with DILI (represented by the graph at the right of step 4, Figure 1). Figure 4(a) shows the drug distribution across 14 therapeutic categories as defined by Anatomic Therapeutic Class (ATC) (http://www.whocc.no/atcddd/) with the RO3 positive drugs highlighted in red. The enrichment of the RO3 drugs in each therapeutic category was determined by Fisher's exact test. We found that two therapeutic categories (i.e., anti-infective for systemic use and musculoskeletal system drugs) were significantly associated with drugs that cause DILI (p-value = 5.00E-11 and 0.002, respectively). To confirm the findings, we carried out the same analysis for drugs in the SIDER database that met the RO3. As shown in Figure 4(b), the same two therapeutic categories were also found to be significantly associated with drugs that cause DILI (p-value = 8.94E-8 and 2.36E-7, respectively). Both results demonstrated that care must be taken when drugs are developed with existing targets in these two categories.

The findings are consistent with real-world observations; for example, non-steroidal anti-inflammatory drugs (NSAIDs, a subcategory of anti-infectives for systemic use) are often associated with DILI. A good example is didanosine (Videx®) which is an antiviral drug used to treat human immunodeficiency virus (HIV) infection. On Jan 29^th^, 2010, the FDA notified healthcare professionals and patients about a rare but serious complication in the liver known as non-cirrhotic portal hypertension in patients using the drug. Subsequently, a black box warning was added to the drug label to warn doctors and consumers of this risk. Didanosine can also cause lactic acidosis and severe hepatomegaly with steatosis, and has resulted in several fatal cases (http://dailymed.nlm.nih.gov/dailymed/drugInfo.cfm?id=23496).

### Associating the protein targets with DILI potential via network analysis

It was important to determine if the drug target is related to the drug's likelihood of causing DILI. Accordingly, we investigated the drugs that were RO3 positive from DrugBank in the target space using network analysis as illustrated on the left side of Figure 1, step 4. These drugs were associated with 134 human protein targets. In the network analysis, we considered that two protein targets are directly related (connected with an edge in network analysis) if one or more drugs were associated with both targets.

**Figure 5:**
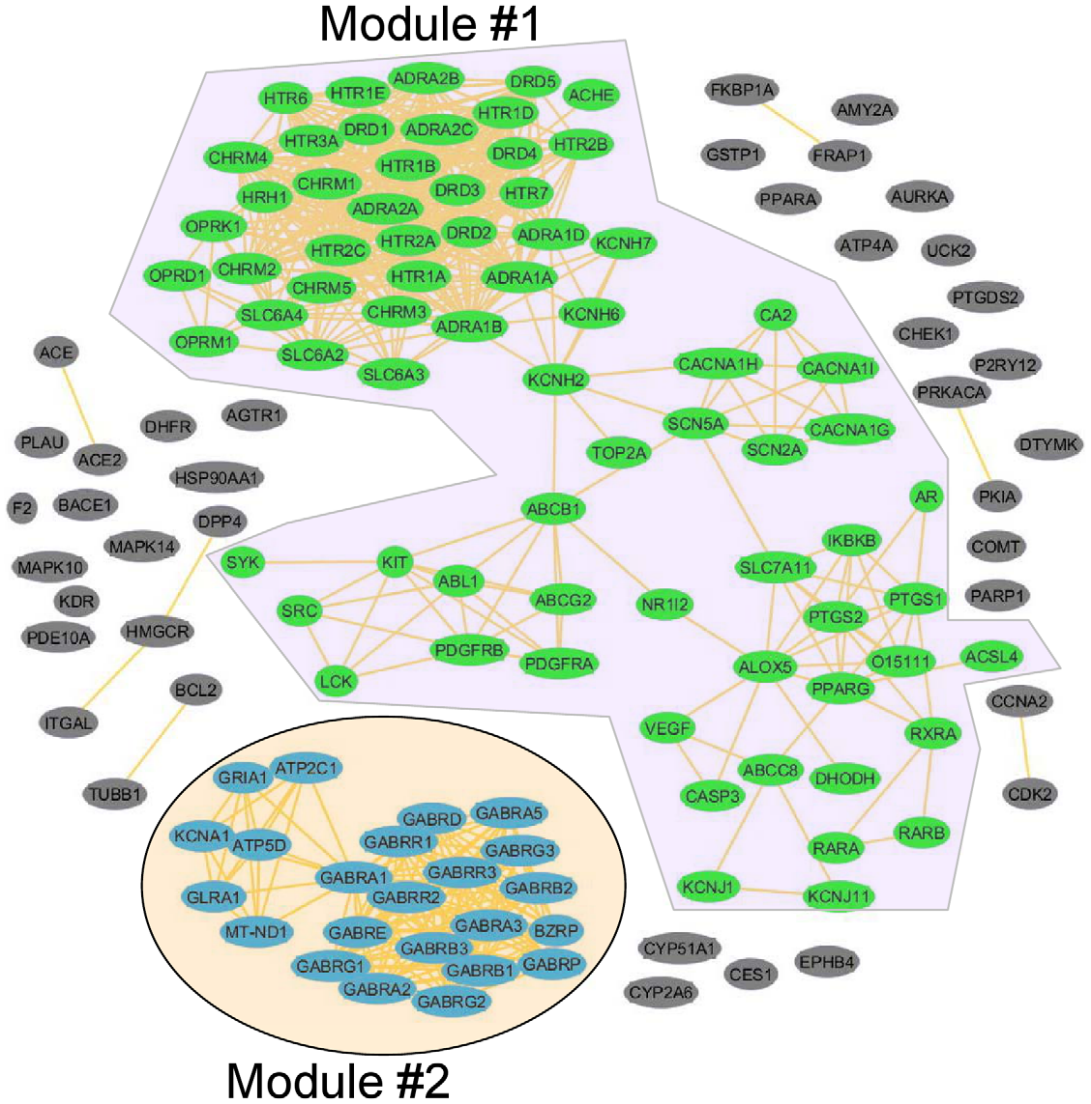
Target network of corresponding drugs satisfying the “Rule of Three”.

As depicted in Figure 5, the network contains two large modules (Modules #1 and #2) with several small modules. There are 72 targets in Module #1 associated with 125 RO3 positive drugs, and 23 targets in Module #2 associated with 8 drugs. We conducted toxicity function and pathway analyses using Ingenuity Pathway Analysis (IPA, http://www.ingenuity.com/) for both modules. In each module, particularly Module #1, the biological functions related to disease and disorder were investigated to assess if the targets of the drugs meeting the RO3 have a relationship with hepatic system diseases or disorders. As shown in Table 3, liver injury and disease related functions enriched in Module #1 were *hepatic system disorder, jaundice, liver cancer, hepatocellular carcinoma, and hepatitis C*. All the liver injury and disease functions are under the hepatic system diseases branch of the top toxicity functions in IPA. The other significant toxicity functions of Module #1 can be found in Supplementary Table S3.

**Table 3:** Summary of significant functions and pathways for module #1 identified in the network analysis of DILI targets using IPA.

Functions Annotation	p-Value	Gene Name	# Genes
hepatic system disorder	5.85E-25	ABCB1, ABCG2, ACSL4, ADRA1A, ADRA1B, ADRA1D, ALOX5, CASP3, CHRM1, CHRM2, CHRM3, CHRM4, CHRM5, CHUK, DRD2, DRD5, HRH1, IKBKB, KIT, NR1I2, OPRM1, PDGFRA, PDGFRB, PPARG, PTGS1, PTGS2, RXRA, SLC6A3, SLC6A4, TOP2A, VEGFA	31
jaundice	2.69E-15	CHRM1, CHRM2, CHRM3, CHRM4, CHRM5, CHUK, HRH1, IKBKB	8
liver cancer	5.58E-11	ABCB1, CA2, CASP3, HTR3A, KIT, LCK, PDGFRA, PDGFRB, PTGS1, PTGS2, RARA, SLC6A3, SLC6A4, SRC, TOP2A, VEGFA	16
hepatocellular carcinoma	7.76E-08	CA2, CASP3, KIT, PDGFRA, PDGFRB, PTGS1, PTGS2, RARA, TOP2A, VEGFA	10
hepatitis C	1.04E-07	CASP3, DRD2, DRD5, OPRM1, PPARG, SLC6A4	6

**Figure 6:**
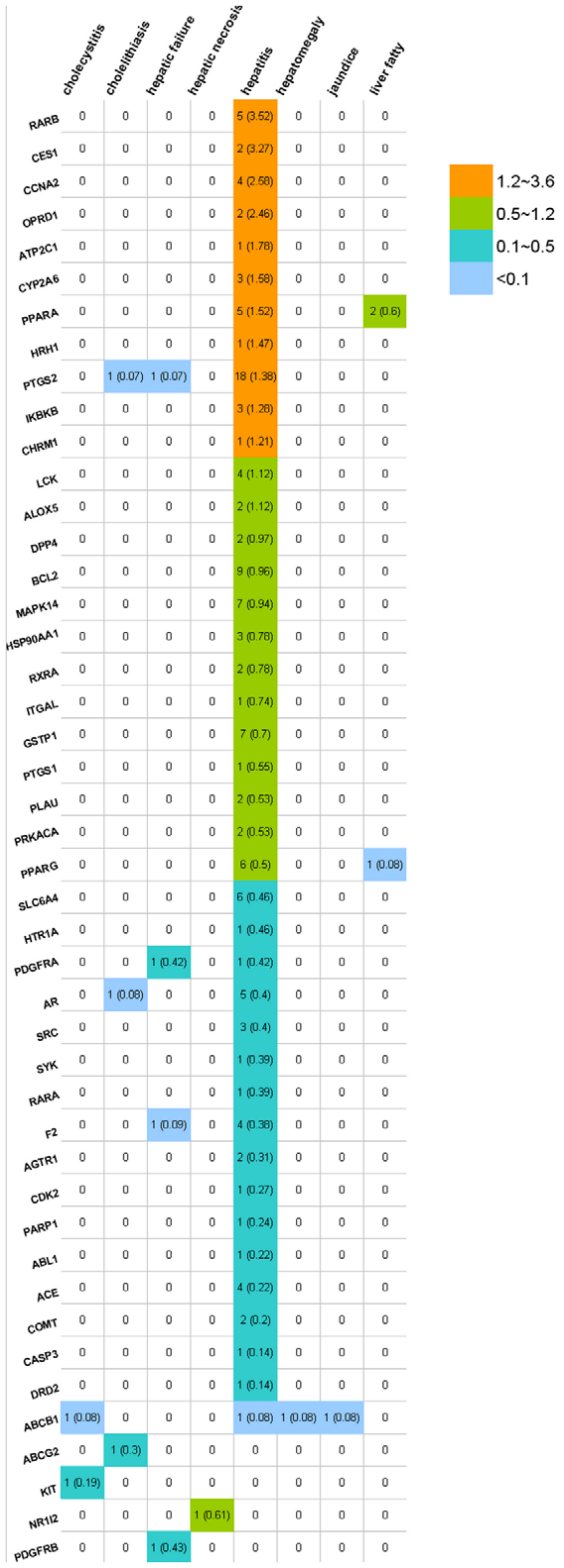
Text mining results to associate types of DILI (columns) with protein targets (rows). The number of co-occurrences (papers) between a target and a side effect type is indicated in the cell. In each cell, the total number of reports as well as the normalized value (shown in parenthesis) is provided. The normalized value is the ratio of the number of co-occurrence reports divided by the total number of reports for a protein target.

We also found that every drug in the two largest modules was associated with more than three targets on average. Note that drugs are prone to having multiple side effects if they interact with multiple targets since different targets may invoke different side effects [16], [17]. We conducted text mining to verify the association of 13 HepSEs and 134 targets identified by RO3 positive drugs. We identified 45 proteins associated with eight HepSEs in a co-occurrence analysis (Figure 6 and Supplementary Table S4). Most of these targets are associated with hepatitis, while targets such as PTGS2/COX-2 (prostaglandin-endoperoxide synthase 2) and ABCD1 (ATP-binding cassette, sub-family, and member 1) are related to multiple HepSEs.

## Discussion

One application of translational science involves utilization of clinical data to address challenges in drug discovery. The key concept of this study is that the side effects observed in clinical trials and post-marketing surveillance can be translated for use in drug discovery. As a proof-of-concept study, we developed the DILIps to address one of the most difficult clinical endpoints to predict from preclinical studies, that is DILI. The DILIps contains three distinct and sequential approaches. First, we identified 13 HepSEs based on the MedDRA ontology, which provided excellent discrimination of a drug's potential to cause DILI (91% and 74% accuracy for LTKB-BD and PfizerData, respectively). Secondly, HepSE-based QSAR models were developed by using all 888 drugs in SIDER, which were highly predictive as compared to published models [14], [18], [19] and offered a robust translation of clinical observation (i.e., side effects) using *in silico* techniques to the drug discovery/preclinical testing aspect of drug development. Next, we developed DILIps by combining these 13 HepSE QSAR models, which yielded 60–70% prediction accuracy for three independent validation sets. Lastly, a RO3 criterion was implemented in DILIps, which had >91% confidence for identification of drugs that might cause severe DILI.

The DILIps is a modular system; each of its components can be replaced by other methods or constructed using different variables. For example, besides selecting 13 HepSEs from the *hepatobiliary disorders* category in MedDRA, we also examined the effect of including additional two DILI related terms from the *investigation* SOC category, or selecting 14 DILI relevant terms as suggested by an expert (Supplementary Table S5). Both yielded similar performance compared to the 13 HepSE-based approach. Given the fact that each MedDRA category is a stand-alone ontology and other options did not yield exceptional performance, we choose the terms under *hepatobiliary disorders* as representative types of DILI in this study. For the second component of the DILIps, we developed HepSE-based QSAR models because chemical structure data were readily available for the entire set of 888 drugs in SIDER, providing a sufficiently large sample from which to build the HepSE-based models. Other technologies, such as gene expression microarrays, might be able to construct better HepSE models. However, the data from these technologies was not available for the complete set of SIDER drugs. With different choices in components 1 and 2, the criterion in component 3 of DILIps could be altered to optimize DILI classification using different consensus approaches instead of RO3. Therefore, the DILIps is subject to change and improvement when new data, technology, and knowledge are available.

Development of predictive models for drugs that might cause DILI in humans has been an active research field, with much of the work being done using QSARs. However, the DILI labels used in these studies are from different sources, some focused on case reports and others developed using text mining. Furthermore, the methods used to develop the models are also different. Thus, it is difficult to compare these methods. For example, Greene *et al*. [14] developed Derek for Windows (DfW), a knowledge-based expert system, to predict a drug's potential to cause DILI using the DILI classification scheme developed by Pfizer. The system has 56% overall accuracy with 73% specificity and 46% sensitivity. Fourches *et al*. [18] applied text mining for DILI reported in different species using MEDLINE abstracts, suggesting that the concordance of liver effects is low (i.e., 39–44%) between different species. They also developed QSAR models using a text mining approach to define DILI classification with external prediction accuracies ranging from 56 to 73%. Very recently, Ekins *et al*. [19] developed a Bayesian model based on DILI endpoint from cellular imaging predictions [7], which gave a concordance of 60%, sensitivity of 56%, and specificity of 67%.

Development of DILI models for humans is always confronted by two distinct but related challenges: (1) a comprehensive drug list with DILI annotation is usually not available, and (2) there is no authoritative assessment of whether a drug causes DILI or not. In this study, we compiled three large datasets from our LTKB project. We used only the drugs of the opposite extremes in DILI classification (positive or negative in relationship to DILI) by removing drugs with ambiguous call. The RO3 criterion of DILIps reached >91% positive predictive value for a combined drug list from these three literature datasets. We also applied DILIps for the drugs with ambiguous call and the results are available from Supplementary Table S6.

The translation of clinical observations to evaluation of drugs earlier in the drug development pipeline is a goal of translational medicine [20]. DILI is an endpoint influenced by several important factors, and it is difficult to adequately predict with a single model. The SIDER database has collected clinical observation data (side effects) from drug labels and the scientific literature, which allows the linkage of disease endpoints and related symptom profiles. This, in turn, provides an opportunity to combine drug information and patient information into a unified prediction method, a focus of this study. The HepSEs provide a new direction to predict DILI based on the consensus of multiple clinical endpoints (side effects) using an *in silico* method.

Elucidation of therapeutic uses, drug targets, and pathways related to DILI from a systematic perspective is of great use in drug discovery and pharmacovigilance. By applying the RO3 criterion to the entire drug space defined by DrugBank, we constructed a DILI landscape in terms of therapeutic and drug target space. We do acknowledge that the findings from this excise are dependent on the accuracy in annotation in DrugBank.

We identified two therapeutic categories (i.e., anti-infectives for systemic use and musculoskeletal system drugs) in which the drugs have a high risk for causing DILI. This is consistent with the general understanding that, for example, NSAIDs (a subcategory of anti-infectives for systemic use) are often associated with DILI and have been subject to a broad range of studies looking into drug-specific, therapeutic class-specific, and genetic-specific effects [21]. Another possibility is that these drugs may have higher exposure rates; they are widely used by many people over prolonged periods, which may inadvertently increase the risk of DILI. The RO3 positive criterion was able to identify most “bad actors” among NSAIDs including celecoxib, diclofenac, diflunisal, ibuprofen, leflunomide, and rofecoxib. Most of them are PTGS2 (COX-2) protein inhibitors. This gene is also involved in several hepatic system pathways such as *hepatic system disorder*, *liver cancer*, and *hepatocellular carcinoma*. COX (Cyclooxygenase) is an enzyme that is responsible for formation of important biological mediators called prostanoids. Pharmacological inhibition of COX can provide relief from symptoms of inflammation and pain. However, more and more reports indicated that the selective inhibition profile of COXs can cause certain serious adverse drug reactions. A classic example is rofecoxib (brand name Vioxx®), which was withdrawn in 2004 because of the risk of heart attack caused by selective inhibition of COX-2. Rofecoxib was also associated with DILI [22]. Another example is lumiracoxib, a selective COX-2 inhibitor developed for the symptomatic treatment of osteoarthritis and acute pain. Concern over hepatotoxicity has contributed to the withdrawal or non-approval of lumiracoxib in most major drug markets worldwide [23]. Therefore, the study of the relationship between drug target and DILI, such as COX selectivity and DILI, may provide new insights into DILI at a molecular level [24].

We also found that DILI drugs often involve multiple targets, which is often associated with drugs applied in multiple therapeutic categories [25]. Drugs interacting with multiple targets are considered “dirty” since they have a potential to initiate different adverse reactions. On the other hand, these drugs may also hold the potential to be repositioned for use outside of their original therapeutic indications. One such example is diclofenac, which is used to relieve pain, tenderness, swelling and stiffness caused by osteoarthritis, rheumatoid arthritis, and ankylosing spondylitis. Diclofenac is labeled with four different ATC codes (i.e., four different therapeutic uses) and associated with a number of targets categorized by DrugBank, including prostaglandin G/H synthase 1 and 2, the cytochrome P450 family (2C18/2E1/2C19/1A2/2C8/2D6/2C9/3A4/1A1/2B6), the UDP-glucuronosyltransferase family (1–1,2B7), prostaglandin G/H synthase 1, etc. Several case-control studies have been carried out to investigate the role of polymorphisms in the gene encoding regions of the aforementioned drug-metabolizing enzymes and transporters to determine susceptibility to diclofenac-induced hepatotoxicity [26], [27], [28], [29], [30]. Diclofenac has been withdrawn in several countries due to liver injury and other adverse drug reactions, including ulcers, bleeding, and ulcerations in the stomach and intestinal linings [31]. Diclofenac induced liver injury causes a number of side effect patterns, including cirrhosis, hepatic failure, hepatic necrosis, hepatitis, jaundice, all of which were included in our set of 13 HepSEs.

DILI is associated with two distinct but related parameters: drug properties and patient susceptibility. Some drugs are more likely to cause DILI, while some patients are more likely to show DILI. The DILIps is primarily capable of addressing the former challenge with an aim to enhance DILI identification in drug discovery. Identifying genetic variations and their associated protein products that contribute to DILI is another important research area, but one that requires the costly and time-consuming collection of samples from large numbers of affected individuals. Study of the genetic risk factors to DILI or other conditions usually involves the identification of genes associated with key disease mechanisms and immunological reactions using genotyping approaches. The network analysis conducted in this study connected DILI drugs with pathways and targets and might contribute to the identification of mechanisms that relate a patient's genetic predisposition and DILI. There are a small number of genetic risk factors identified for DILI, most are associated with a drug interaction with a specific HLA (human leukocyte antigen system) polymorphism within the major histocompatibility complex (MHC) such as lumiracoxib (HLA-DRB1*15∶01) [23], antituberculosis chemotherapy (HLA-DQB1*02∶01) [32], ticlopidine (HLA-A*33∶03) [33], ximelagatran (HLA-DRB1*07∶01) [34], flucloxacillin (HLA-B*57∶01) [21], and amoxicillin-clavulanate (HLA-DRB1*15∶01) [35]. Other genetic risk factors such as those involving drug metabolizing enzymes are exemplified by CYP2C8*4 (diclofenac), CYP2E1*1A (isoniazid), *GSTT1-M1* (troglitazone), and UGT2B7*2 (diclofenac) are also reported [36], [37], [38].

Drug safety is a key area of focus in the FDA. Modernizing safety evaluation has been advocated by the FDA's recent initiative on advancing regulatory science with a proposal of incorporating both *in vitro* and *in silico* methodologies in drug development and safety assessment [39]. The DILIps follows the same philosophy that underlies this new initiative at the FDA. It could be a predictive system for FDA to utilize and reference when hepatotoxicity issues arise during the various stages of the regulatory review process. It could also serve as a proof-of-concept approach of using predictive systems for drug safety to support the FDA's regulatory science. While the DILIps was developed for DILI, its methodology can be applied equally well to address other drug safety issues, such as renal and cardiovascular toxicity.

## Materials and Methods

### Preparation of datasets

#### SIDER database

SIDER is computer-readable database of side effects which connects 888 drugs with 1450 different side effect terms [12]. The side effects were extracted from drug labels in either Structured Product Labeling (SPL) or Portable Document Format (PDF) documents. The standardized Coding Symbols for a Thesaurus of Adverse Reaction Terms (COSTART), a part of the Unified Medical Language System (UMLS) Metatheasaurus, was used as the basic lexicon of side effects. In this study, we downloaded the entire database from http://sideeffects.embl.de/. We then constructed a matrix with 888 drugs corresponding to 1450 side effects with supplementing the chemical structure data.

#### DrugBank

DrugBank (http://www.drugbank.ca) is a richly annotated database of drugs and drug target information [40], [41]. It contains extensive information about nomenclature, chemistry, structure, function, mode of action, pharmacology, pharmacokinetics, metabolism, and pharmaceutical properties of both small molecule and large molecule (biotech) drugs. The updated DrugBank 3.0 contains 6,800 drug entries including 1,400 FDA-approved small molecule drugs, 132 FDA-approved biotech (protein/peptide) drugs, 82 nutraceuticals and 5,200 experimental drugs. In additional, more than 4,300 non-redundant protein (i.e. drug target) sequences are linked to these drug entries [42].

In this study, information about 6620 small molecule drugs (1,400 FDA drugs and 5,200 experimental drugs) was retrieved including chemical structure, approval status, therapeutic categories and protein targets for use to generate the DILI landscape in terms of therapeutic uses and drug targets.

#### LTKB benchmark dataset (LTKB-BD)

As a part of the LTKB project, a research team from the FDA's National Center for Toxicological Research has developed the LTKB-BD dataset that contains 287 drugs with DILI annotation based on the FDA-approved drug labels. The data are available from http://www.fda.gov/ScienceResearch/BioinformaticsTools/LiverToxicityKnowledgeBase/ucm226811.htm [13]. The drugs are classified into three categories: those of Most-DILI-Concern, Less-DILI-Concern, and No-DILI-Concern. In this study, only those in the Most-DILI-Concern (gemtuzumab was excluded since it is a biotechnology product) and No-DILI-Concern categories were used. The dataset was divided into two sets. One set overlapped with the SIDER database and contained 69 drugs of Most-DILI-Concern and 59 No-DILI-Concern. This was used to evaluate the performance of HepSEs. The rest of the LTKB-BD contained 67 drugs of Most-DILI-Concern and 6 of No-DILI-Concern that were not in SIDER and were used to validate the DILIps performance (Supplementary Table S6).

#### Pfizer hepatotoxicity dataset (PfizerData)

Another independent test set comes from part of the Derek for Windows (DfW) system [14], which is a knowledge-based expert system designed to assess the potential toxicity of a chemical from its structure. A total of 626 compounds were classified into four categories based on case reports, including evidence of human hepatotoxicity (HH), no evidence of hepatotoxicity in any species (NE), weak evidence (<10 case reports) of human hepatotoxicity and evidence for animal hepatotoxicity but not tested in humans. In this study, only HH and NE drugs were used, As a result, there were 406 drugs remaining; 168 HH (positive) and 90 NE (negative) overlapped with the SIDER database. The other 92 HH (positive) and 56 NE (negative) that were not contained in the SIDER database were selected as another independent test set (Supplementary Table S6).

#### O'Brien et al. dataset

O'Brien et al. classified drugs into four categories according to the severity of human hepatotoxicity based on the frequency of an observed increase in ALT and other evidence [15]. In this study, the categories of “Severely” and “Moderately” hepatotoxic drugs were considered DILI positive drugs while non-toxic drugs were considered DILI negative, and those that did not overlap with the SIDER database were employed. The ratio of positive to negative drugs was 25/15 (Supplementary Table S6).

### Data analysis method

#### Identification and assessment of Hepatotoxic Side Effects (HepSEs)

This section is shown as step 1 in Figure 1. There are 1450 different side effects listed in the SIDER database. We identified 473 side effects for HepSE identification, with each side effect associated with more than 20 drugs. We used MedDRA to identify HepSEs. MedDRA is an ontology that provides a controlled vocabulary describing adverse events. The 473 side effect terms were mapped to the System Organ Class (SOC) level of *hepatobiliary disorders* in MedDRA to extract the HepSEs (Supplementary Table S1). The drugs in LTKB-BD which overlapped with drugs in SIDER (128 total drugs) as well as those in PfizerData which overlapped with drugs in SIDER (258 total drugs) were employed to assess the performance of HepSEs. If a drug was associated with any HepSE as observed in the SIDER database, it was considered as DILI positive. To determine if the predictive performance of the 13 HepSE models was better than would be expected by chance alone we randomly selected a set of *M* side effects (*M* = 3,5,…,21) and used these to predict DILI potential. The selection process for each *M* was repeated 20,000 times, and the average performance of each *M* was compared to the performance of the 13 HepSEs.

#### DILI prediction system (DILIps)

Development of DILIps consists of two steps (steps 2 and 3 of Figure 1). In step 2, all of the drugs were transformed into well-established functional class fingerprints (FCFP_6), structural fingerprint developed by Pipeline Pilot 8.0 from SciTegic (http://accelrys.com/). It has been shown in other studies that Bayesian models built using circular fingerprints work very well in virtual screening tasks [14], [43], [44], [45], [46], [47]. Then, multiple-category naïve Bayesian classifiers were trained for each of the selected HepSE endpoints. In the training set, leave-one-out cross-validation (LOO-CV) was employed to investigate the model performance. For each model, a receiver operating characteristic plot (ROC plot) was drawn to select the best Bayesian score (cut-off value) to distinguish DILI drugs and non-DILI drugs.

In step 3, the independent test sets were submitted to the 13 HepSE models to calculate the Bayesian scores and give the prediction results: For each HepSE endpoint, the predicted Bayesian scores (PB-SCORE*_i_*, *i* = 1, 2…*n*) compared to cut-off Bayesian score obtained in step 2. If PB-SCORE*_i_* > cut-off value, the drug was considered positive for this endpoint and vice versa. A drug was considered to have the potential to cause DILI if any of the HepSE endpoints was called as positive (the left side of Figure 1, step 3). In the right side of Figure 1 (step 3), consensus prediction strategies were used to investigate the effectiveness of combining results from multiple HepSEs into a single prediction. A “Rule of N” strategy was evaluated, where 13 separate consensus prediction strategies were examined with each predicting a drug as causing DILI if N (N = 1,2,…,13) HepSEs were positive for that drug.

#### DILI landscape

Three sets of analysis were conducted, which is summarized in step 4 of Figure 1. The right side of Figure 1 is to assess the relationship of therapeutic use and DILI potential of RO3 drugs. The Anatomical Therapeutic Chemical (ATC) codes [48] for small molecule drugs which meet the “Rule of Three” were extracted for this analysis.

The right side of Figure 1 is to assess the association of protein targets and DILI potential of RO3 drugs. The protein targets associated with small molecule drugs which meet the RO3 criterion were obtained from DrugBank 3.0. There are 4437 different protein targets from different organisms, and only the human protein targets were selected. The protein target network was built by considering two protein targets as connected if at least one drug was associated with both targets. Two large modules were identified using the SCAN algorithm, which is used to find modules in the network [49]. The protein targets in these two large modules were submitted to Ingenuity Pathway Analysis (IPA) software (http://www.ingenuity.com/products/pathways_analysis.html) for pathway analysis. In addition, a text mining with co-occurrence analysis [50] was also employed to verify the protein target and HepSE relationship from the network analysis. In this analysis, the number of papers in PubMed that links a target with a HepSE in a co-occurrence analysis was extracted. Since some proteins are more extensively studied than others, the number of papers associating the protein to the HepSE was normalized by dividing the number of co-occurrence reports by the total number of reports of the related protein.

## Supporting Information

Figure S1 **The distribution of 888 drugs over 473 side effects and vice versa.**
(TIF)

Table S1 **Information for the 473 side effects.** Information included: (1) the number of drugs involved; (2) system organ classes (SOC) number and annotation of MedDRA; and (3) the distribution of 473 side effects in the SOC levels of MedDRA.
(XLS)

Table S2 **Information of the validation set from three literature datasets (i.e., LTKB-BD, PfizerData, and O'Brien et al.) for drugs that do not overlap with SIDER.**
(XLS)

Table S3 **The top toxicity functions of Module 1.**
(XLS)

Table S4 **The literature proof about the co-occurrence between the HepSE terms and protein target.** The EntrezGene ID and PubMed ID (which can be linked to PubMed directly) are provided.
(XLS)

Table S5 **The QSAR models performance for the 13 HepSEs and an additional two terms from the** ***Investigation*** **category, and for 14 HepSEs as suggested by an expert.**
(XLS)

Table S6 **Information for datasets.** Datasets include: (1) SIDER, (2) LTKB-BD, (3) PfizerData, (4) O'Brien et al., and (5) Small molecules in DrugBank.
(XLS)
