# Supplementary figures and images for: Translating Clinical Findings into Knowledge in Drug Safety Evaluation - Drug Induced Liver Injury Prediction System (DILIps)
Source: PLoS Comput Biol. 2011 Dec 15;7(12):e1002310. doi: 10.1371/journal.pcbi.1002310 (PMC3240589; doi:10.1371/journal.pcbi.1002310)

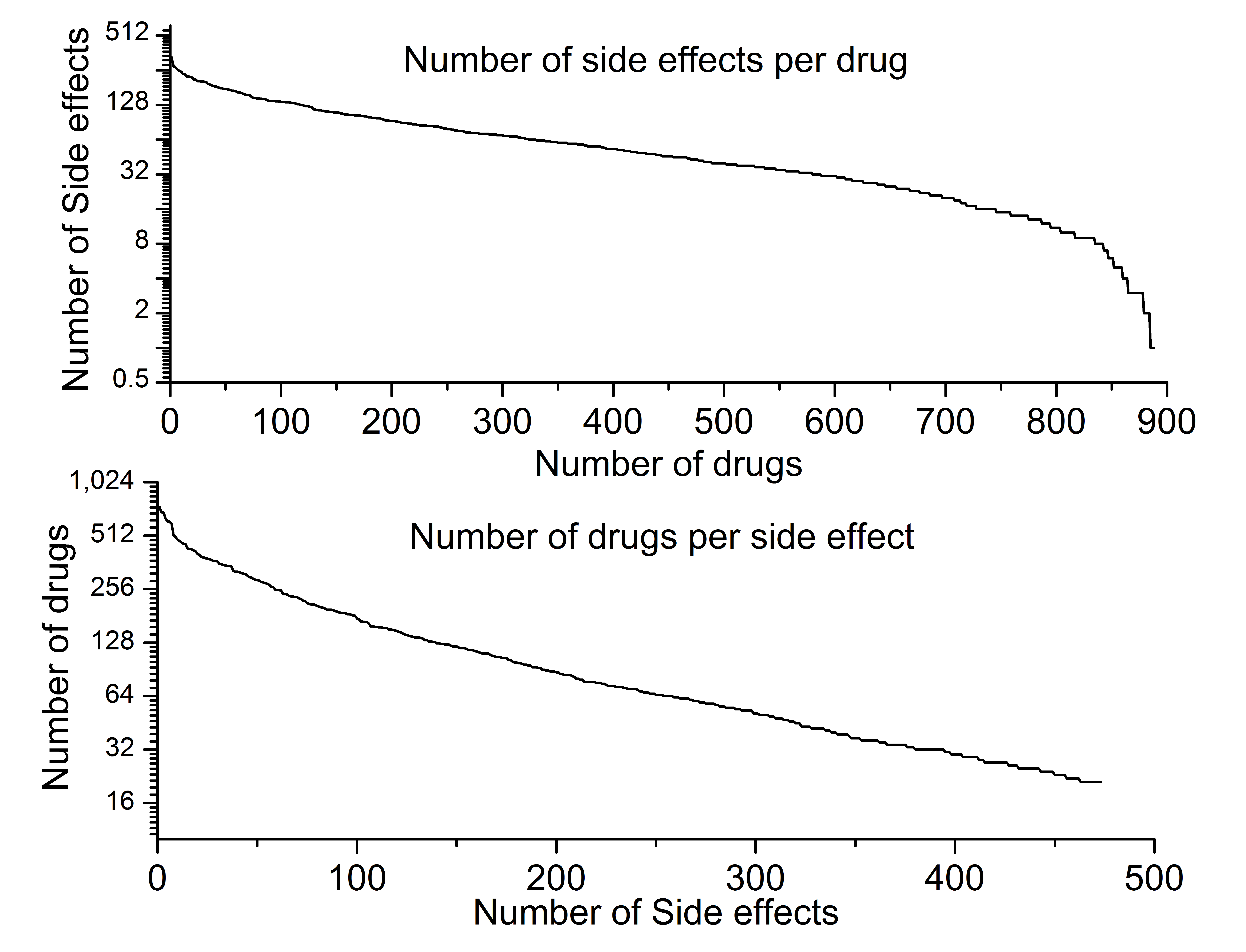

Supplement: Figure S1 — The distribution of 888 drugs over 473 side effects and vice versa. (TIF) [file pcbi.1002310.s001.tif]
